# Functional Alterations in Ciliogenesis-Associated Kinase 1 (CILK1) that Result from Mutations Linked to Juvenile Myoclonic Epilepsy

**DOI:** 10.3390/cells9030694

**Published:** 2020-03-12

**Authors:** Eric J. Wang, Casey D. Gailey, David L. Brautigan, Zheng Fu

**Affiliations:** 1 Department of Pharmacology, University of Virginia School of Medicine, Charlottesville, VA 22908, USA; ew8sk@virginia.edu (E.J.W.); cdg7sf@virginia.edu (C.D.G.); 2 Department of Microbiology, Immunology, and Cancer Biology, University of Virginia School of Medicine, Charlottesville, VA 22908, USA; db8g@virginia.edu; 3 NCI-Designated Cancer Center, Cancer Biology Program, University of Virginia School of Medicine, Charlottesville, VA 22908, USA

**Keywords:** cilia, ciliopathy, ciliogenesis, epilepsy, kinase, phosphorylation

## Abstract

Ciliopathies are a group of human genetic disorders associated with mutations that give rise to the dysfunction of primary cilia. Ciliogenesis-associated kinase 1 (CILK1), formerly known as intestinal cell kinase (ICK), is a conserved serine and threonine kinase that restricts primary (non-motile) cilia formation and length. Mutations in CILK1 are associated with ciliopathies and are also linked to juvenile myoclonic epilepsy (JME). However, the effects of the JME-related mutations in CILK1 on kinase activity and CILK1 function are unknown. Here, we report that JME pathogenic mutations in the CILK1 N-terminal kinase domain abolish kinase activity, evidenced by the loss of phosphorylation of kinesin family member 3A (KIF3A) at Thr672, while JME mutations in the C-terminal non-catalytic domain (CTD) have little effect on KIF3A phosphorylation. Although CILK1 variants in the CTD retain catalytic activity, they nonetheless lose the ability to restrict cilia length and also gain function in promoting ciliogenesis. We show that wild type CILK1 predominantly localizes to the base of the primary cilium; in contrast, JME variants of CILK1 are distributed along the entire axoneme of the primary cilium. These results demonstrate that JME pathogenic mutations perturb CILK1 function and intracellular localization. These CILK1 variants affect the primary cilium, independent of CILK1 phosphorylation of KIF3A. Our findings suggest that CILK1 mutations linked to JME result in alterations of primary cilia formation and homeostasis.

## 1. Introduction

The primary cilium is a single diminutive protrusion on the apical surface of almost all vertebrate cells that is enriched in receptors, ion channels, second messengers, and various signaling pathways. It employs these factors to sense and transduce environmental and hormonal signals to regulate intracellular processes and control cell behaviors [1]. The primary cilium is a dynamic structure that is resorbed and reassembled during each cell division cycle and it plays an essential role in tissue development and homeostasis. Defects in primary cilium function are linked to human diseases collectively called ciliopathies [2]. The mechanisms underlying the pathological phenotypes associated with ciliopathies are still poorly understood.

Ciliogenesis-associated kinase 1 (CILK1) [3], formerly known as intestinal cell kinase (ICK) and MAK-related kinase (MRK) [4,5] are broadly expressed in human and mouse tissues. CILK1 is closely related to male germ cell-associated kinase (MAK) [6,7] and they cluster with MAK/MRK/MAPK-overlapping kinase (MOK) [8] in the *v-ros* cross-hybridizing kinase (RCK) family of protein kinases. CILK1 is the prototype for this group of kinases that have similarities with both mitogen-activated protein kinases (MAPKs) and cyclin-dependent protein kinases (CDKs) in the N-terminal catalytic domain. CILK1 is activated by phosphorylation of its MAPK-like TDY motif in the activation loop by an upstream activating kinase CDK20 (cyclin-dependent kinase 20), also known as CCRK (cell cycle-related kinase) [9,10]. CILK1 is partially inactivated by phosphorylation of its CDK-like regulatory site Tyr15 near the N-terminus [11]. CILK1 has a long and intrinsically unstructured non-catalytic C-terminal domain (CTD) that is critical for substrate binding and cilia targeting [12]. Candidate substrates for CILK1, such as KIF3A [12,13], Scythe [10,14] and mTORC1 [15], have emerged.

RCK kinases and their homologs, including *C. elegans* DYF5 (dye-filling defective 5), *Chalmydomonas* LF4 (long flagella protein 4), *Tetrahymena* LF4A, and *Leishmania* LmxMPK9 (*L. mexicana* MAP kinase 9), are conserved modulators of cilium and flagellum length [14,16,17,18,19,20,21,22,23]. Inactivating mutations in human *CILK1*, including c.1305G-A (R272Q), c.358G-T (G120C) and c.238G-A (E80K), are associated with human ciliopathies [23,24,25]. Both *Cilk1* R272Q knock-in [14,26] and *Cilk1* knockout [13,22] mouse models have reproduced human ciliopathy phenotypes. Bidirectional intraflagellar transport (IFT) along the microtubule bundles in the primary cilium involves kinesin-mediated anterograde and dynein-mediated retrograde movement. Anterograde IFT is critical for proper cilium formation and maintenance [27]. CILK1 knockdown in IMCD-3 cells accelerates anterograde IFT in the primary cilium [20], which may explain CILK1 deficiency-induced cilium elongation [14,20,22,23].

Pathogenic variants of CILK1 were recently linked to juvenile myoclonic epilepsy (JME) [28]. The JME variants of CILK1 impair neuronal cell activities such as mitosis, cell cycle exit, and migration. An important question not yet addressed is whether the JME phenotypes are related to the function of CILK1 in the primary cilium. Here, we demonstrate that CILK1 variants linked to JME have lost or compromised functions in suppressing cilia length but also gained functions in promoting ciliogenesis in NIH-3T3 cells. Our data suggest that the mechanism by which CILK1 variants impact cilia length and ciliogenesis is independent of CILK1 phosphorylation of the kinesin motor protein KIF3A and is related to CILK1 mislocalization in the primary cilium.

## 2. Materials and Methods

### 2.1. Plasmids and Antibodies

pEBG-GST-mCILK1 and pEGFP-mCILK1 plasmids encoding mouse CILK1 wild type (WT), kinase dead (KD), and R272A mutants were described in [5,9]. Human CILK1 in pCMV6-entry (RC216454) from Origene (Rockville, MD, USA) was subcloned into pEBG-GST and pEGFP vectors. GST-hCILK1 and GFP-hCILK1 variants I102L, K220E, K305T, V320I, A615T, and R632X were generated by site-directed mutagenesis. pCMV6-hKIF3A (Myc-FLAG-tagged) (RC221331) was from Origene (Rockville, MD, USA).

GFP (B-2) mouse monoclonal antibody (sc-9996) was from Santa Cruz (Dallas, TX, USA). KIF3A (D7G3) rabbit monoclonal (#8507) and FLAG-tag (D6W5B) rabbit monoclonal (#14793) antibodies were from Cell Signaling Technology (Danvers, MA, USA). Arl13B rabbit polyclonal antibody (17711-1-AP) was from Proteintech (Rosemont, IL, USA). Gamma-Tubulin mouse monoclonal antibody (GTX11316) was from GeneTex (Irvine, CA, USA). Goat anti-rabbit IgG (Alexa Fluor 594-conjugated) antibody (ab150084) and goat anti-mouse IgG (Alexa Fluor 647-conjugated) antibody (ab150115) were from Abcam (Cambridge, MA, USA). KIF3A-phospho-Thr672 antibody was generated in rabbits against phospho-KIF3A peptide RPR[pT]SKGKARPKTGC at GenScript (Piscataway, NJ, USA) and affinity-purified as described in [12].

### 2.2. Cell Culture and Transfection

HEK293T and NIH-3T3 cells were maintained at 37 °C and 5% CO_2_ in Dulbecco’s modified Eagle’s medium (DMEM) supplemented with 4.5 g/L glucose and 10% fetal bovine serum (FBS) or 10% new born calf serum (NBCS). HEK293T cells were transfected using a calcium phosphate protocol as described in [29], and NIH-3T3 cells were transfected using the lipofectamine 2000 reagent following the manufacturer’s instruction.

### 2.3. Immunoprecipitation and Immunoblotting

Forty eight hours after co-transfection of GST-CILK1 and FLAG-KIF3A, HEK293T cells were lysed in lysis buffer (50 mM Tris-HCl, pH 7.4, 150 mM NaCl, 1% NP-40, 2 mM EGTA, complete protease inhibitors (Roche, Basel, Switzerland), 10 mM sodium orthovanadate, 5 mM sodium fluoride, 10 mM sodium pyrophosphate, 10 mM β–glycerophosphate, and 1 µM microcystin LR). Cell lysate was cleared by centrifugation. FLAG-KIF3A proteins were immunoprecipitated from cell lysate using FLAG-tag rabbit monoclonal antibody and captured on GammaBind Sepharose beads (GE Healthcare, Chicago, IL, USA).

Sepharose beads were boiled for 5 min in an equal volume of 2× Laemmli sample buffer (120 mM Tris-HCl, pH 6.8, 4% SDS, 20% glycerol, 10% β-mercaptoethanol, 0.02% bromophenol blue) and loaded on an SDS gel. Samples were transferred to a polyvinylidene fluoride (PVDF) membrane and blocked for one hour in 5% dry milk before primary antibody incubation in Tris-buffered saline (TBS) containing 0.1% Tween-20 and 5% bovine serum albumin (BSA) overnight at 4 °C. This was followed by extensive rinses and one-hour incubation with horseradish peroxidase (HRP)-conjugated secondary antibody. Chemiluminescence signals were developed using Millipore Immobilon enhanced chemiluminescence (ECL) reagents (EMD Millipore, Burlington, MA, USA).

### 2.4. Confocal Immunofluorescence Microscopy and Imaging

NIH-3T3 cells grown on gelatin-coated coverslips were fixed by 4% paraformaldehyde (PFA) in PBS, rinsed in PBS, and then permeabilized by 0.2% Triton X-100 in PBS. After one hour in blocking buffer (3% goat serum, 0.2% Triton X-100 in PBS), NIH-3T3 cells on cover slips were incubated with basal body marker Gamma-Tubulin mouse antibody (GeneTex, Irvine, CA, USA, GTX11316) and/or cilia marker Arl13B rabbit antibody (ProteinTech, Rosemont, IL, USA, 17711-1-AP) at 4 °C overnight followed by rinses in PBS and one hour incubation with goat anti-mouse IgG (Alexa Fluor 647-conjugated) secondary antibody (Abcam, Cambridge, MA, USA, ab150115) and/or goat anti-rabbit IgG (Alexa Fluor 594-conjugated) secondary antibody (Abcam, Cambridge, MA, USA, ab150084). After extensive rinses, slides were mounted in antifade reagent containing DAPI (4′,6-diamidino-2-phenylindole) for imaging via ZEISS LSM 700 Confocal Microscope (Carl Zeiss, Jena, Germany).

The Zen 2009 program was used with the confocal Laser Scanning Microscope (LSM) 700 to collect z stacks at 0.5 μm intervals to incorporate the full axenome based on cilia marker Arl13b staining. All cilia were then measured in Fiji/ImageJ (v1.52p) via a standardized method based on the Pythagorean Theorem, as described in [30]. Analysis of cilia length was based on the equation *L*^2^ = *z*^2^ + *c*^2^, in which “c” is the longest flat length measured of the z slices and “z” is the number of z slices in which the measured cilia was present multiplied by the z stack interval (0.5 μm).

For GFP-positive cells, the average pixel intensity between zero (no signal) to 255 (maximum signal) was measured of GFP channel using ImageJ to create an averaged GFP intensity score. Cells that highly overexpressed GFP at pixel values above 120 were excluded from analysis. Control cells expressed GFP at pixel values lower than five. Cells expressing moderate levels of GFP at pixel values in the range from 20 to 70 were selected for analysis of primary cilia (Figure S2). Ciliation measurements were based on analyses of multiple randomized fields of 2–20 cells, excluding any cell that was mitotic or highly overexpressed GFP. For ciliated cells in these fields, the cilia length and localization pattern were recorded for statistical analysis.

### 2.5. Statistical Analysis

Quantified experimental data were analyzed by the Student *t*-test. Data were reported as mean ± standard deviation (SD). *P*-values less than 0.05 were considered as significant.

## 3. Results

### 3.1. CILK1 Variants Lack Restrictive Effects on Cilia Length

**Figure 1:**
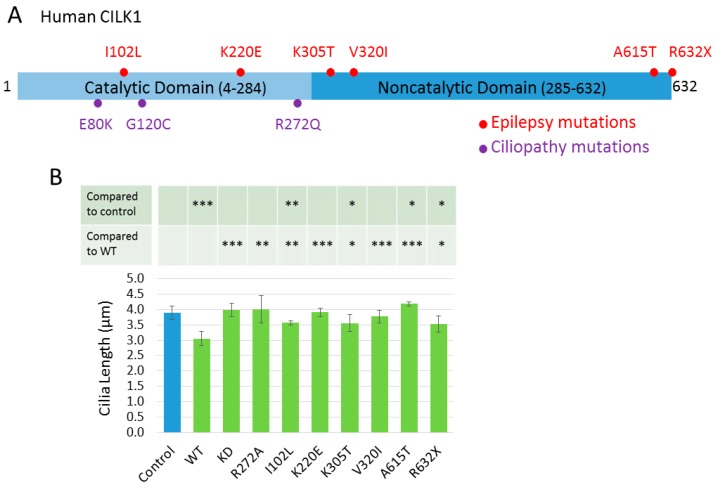
(**A**) Human ciliogenesis-associated kinase 1 (CILK1) protein domain structure and the pathogenic variants identified in human ciliopathies [23,24,25] and epilepsy [28]. Human CILK1 has two basic structural domains: the N-terminal catalytic domain (4-284 aa) and the C-terminal non-catalytic domain (285-632 aa), which is an intrinsically disordered protein region with critical functions in CILK1 substrate binding and cilia localization. Three pathogenic mutations (E80K, G120C, and R272Q) in the catalytic domain are associated with ciliopathies. Six pathogenic or likely pathogenic variants located in both the catalytic (I102L and K220E) and the non-catalytic (K305T, V320I, A615T, and R632) domains are associated with juvenile myoclonic epilepsy. (**B**) Effects of CILK1 variants on cilia length. GFP-CILK1 (wild type (WT); kinase-dead (KD); inactive mutant R272A; juvenile myoclonic epilepsy (JME) variants) were expressed in NIH-3T3 cells. The primary cilium was immunostained with rabbit anti-Arl13B. Cilia length was measured in GFP-CILK1 positive cells (n = 540) and control cells (n = 540). Shown are mean ± SD, *** *P* < 0.001, ** *P* < 0.01, * *P* < 0.05.

Mutations of CILK1 found in JME [28] and ciliopathies [23,24,25] are scattered across the N-terminal catalytic domain (NTD) and the C-terminal non-catalytic domain (CTD) (Figure 1A). Overexpression of CILK1 in NIH-3T3 [23] and IMCD-3 [20] cells negatively regulates cilia length, so we tested whether CILK1 variants found in JME retain this ability to restrict cilia length. We transfected various GFP-CILK1 constructs into NIH-3T3 cells and immunostained the primary cilium with anti-Arl13B. GFP-CILK1 wild type and variants were expressed at similar levels when normalized against β-actin, based on Western blotting (Figure S1). GFP-positive cells expressing moderate GFP fluorescence at pixel values in the range from 20 to 70 were selected for analysis (Figure S2). Control cells were either not transfected or were transfected but expressed GFP-CILK1 at pixel values below five (Figure S2). We used confocal immunofluorescence microscopy to image primary cilia and measured cilia length of both GFP-positive and control cells. The average cilium length of GFP-positive cells expressing CILK1 variants K220E and V320I was not significantly shorter than that of control cells (Figure 1B), indicating that variants K220E and V320I did not restrict cilia length, in contrast to WT GFP-CILK1. On the contrary, GFP-positive cells expressing CILK1 variants I102L, K305T and R632X exhibited shorter cilia when compared with control cells, but their restrictive effect on cilia length was significantly less than that of WT GFP-CILK1 (Figure 1B), indicating that variants I102L, K305T, and R632X have compromised ability to restrict cilia length. We noted that GFP-positive cells expressing the A615T variant exhibited slightly longer cilia than control cells (Figure 1B). As controls, cells expressing either the kinase dead (KD, artificially made mutation K33R) or the inactive mutant (R272A) form of GFP-CILK1 showed no significant change in cilia length (Figure 1B). Given the variation in cilia length of control cells associated with each variant, we also presented the above data in Figure S3, showing the impact of CILK1 variants on cilia length relative to each individual control. These data together show that the pathogenic variants of CILK1 found in JME lose the ability to restrict length of the primary cilium.

### 3.2. Phosphorylation of KIF3A by CILK1 Variants Found in Human JME

**Figure 2:**
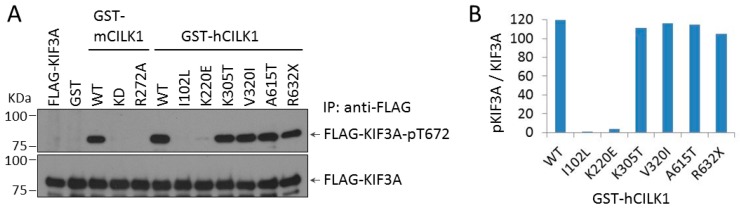
(**A**) Effects of CILK1 variants on phosphorylation of FLAG-kinesin family member 3A (KIF3A) Thr672. GST or GST-CILK1 (wild type (WT); kinase-dead (KD); disease variants) was co-expressed with FLAG-KIF3A in HEK293T cells. Total and phospho-Thr672-specific signals of FLAG-KIF3A were assessed by immunoblotting of anti-FLAG immunoprecipitates with anti-pKIF3A-Thr672 and anti-KIF3A, respectively. (**B**) Quantification of FLAG-KIF3A-phospho-Thr672 signals normalized against total FLAG-KIF3A signals in (**A**).

We sought to examine whether these variant CILK1 can phosphorylate KIF3A. We co-expressed human GST-CILK1 with FLAG-KIF3A in cells and assayed phosphorylation of FLAG-KIF3A at Thr672 by immunoblotting (Figure 2). GST-hCILK1 variants (I102L and K220E) in the NTD did not support FLAG-KIF3A phosphorylation at Thr672, whereas other GST-hCILK1 variants (K305T, V320I, A615T, and R632X) in the CTD were not much different from WT in terms of KIF3A-Thr672 phosphorylation. As controls, GST-mCILK1 WT, but not kinase-dead (KD), nor the inactive mutant (R272A), phosphorylated FLAG-KIF3A at Thr672. Expression of GST alone did not support phosphorylation of FLAG-KIF3A. These results indicate that CILK1 variants associated with JME exhibited compromised function in restricting cilia length but segregated into those that did or did not phosphorylate KIF3A.

### 3.3. CILK1 Variants Gain the Ability to Promote Ciliogenesis

**Figure 3:**
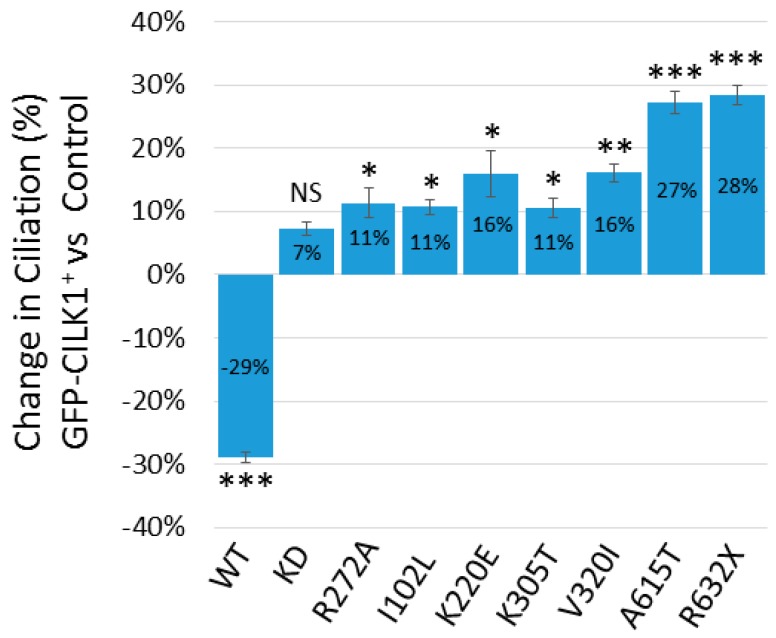
Effects of CILK1 variants on ciliogenesis. GFP-CILK1 (wild type (WT); kinase-dead (KD); disease variants) were expressed in NIH-3T3 cells. The primary cilium was immunostained with anti-Arl13B. Ciliated cells in both GFP-CILK1-positive and control populations were counted under a confocal immunofluorescence microscopy. Shown is the change in ciliation % of GFP-positive cells (n = 120 cells) relative to control cells (n = 120 cells). Mean ± SD, * *P* < 0.05, ** *P* < 0.01, *** *P* < 0.001, NS = not significant.

We examined the effect of these mutations in CILK1 on the ability to regulate ciliogenesis, by assessing the percentage of ciliated individual cells in NIH-3T3 cells expressing GFP-CILK1. We scored the percentage of GFP-positive cells versus control cells with a primary cilium and found expression of GFP-CILK1 WT reduced ciliation by about 30% (Figure 3). In contrast, CILK1 variants exhibited no reduction in ciliogenesis (Figure 3); instead, both an inactive mutant (R272A) and epilepsy variants of CILK1 showed significant increases in ciliation. Notably, the A615T and R632X variants increased ciliation of cells by almost 30%. The two variants (A615T and R632X) that have the most profound effect in promoting ciliogenesis retained the ability to phosphorylate KIF3A. On the other hand, the KD mutant does not phosphorylate KIF3A and has no significant stimulatory effect on ciliogenesis (Figure 3). These results revealed the unexpected properties of CILK1 variants—that variants which had compromised ability to restrict cilia length could also gain function in promoting ciliogenesis.

### 3.4. CILK1 Variants Are Mis-Localized Along the Axoneme of the Primary Cilium

**Figure 4:**
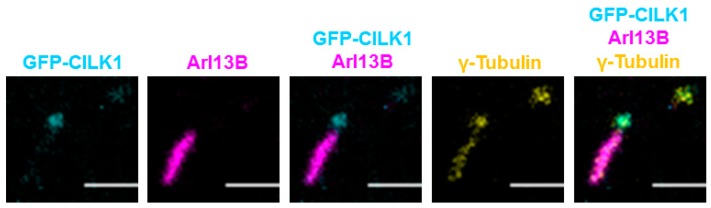
GFP-CILK1 localizes to the base of the primary cilium. NIH-3T3 cells were transfected with GFP-CILK1 and immunostained for the cilium and the centriole with anti-Arl13B and anti-γ-tubulin, respectively. Pseudocolors for GFP-CILK1 (cyan), Arl13B (magenta), and γ-tubulin (yellow) were used to facilitate visualization. Scale bar, 2 µm.

We co-immunostained the basal body and the primary cilium in NIH-3T3 cells with anti-γ-tubulin and anti-Arl13B and visualized the localization of GFP-CILK1 WT (Figure 4). The images revealed that GFP-CILK1 is concentrated at the basal body. This is seen by the overlapping of GFP-CILK1 with γ-tubulin staining of the basal body, but separation of GFP-CILK1 from Arl13B staining of the cilium axoneme (Figure 4). It is worth pointing out that the gamma-tubulin localization in the cilium shaft was likely a bleed-through artifact due to crossover of fluorescence emission from channel 594 into 647. There is a significant overlap between the spectral profiles of fluorophores 594 and 647. In an experiement involving a single fluorophore labelling, γ-tubulin staining only occurred in the centrioles (data not shown).

**Figure 5:**
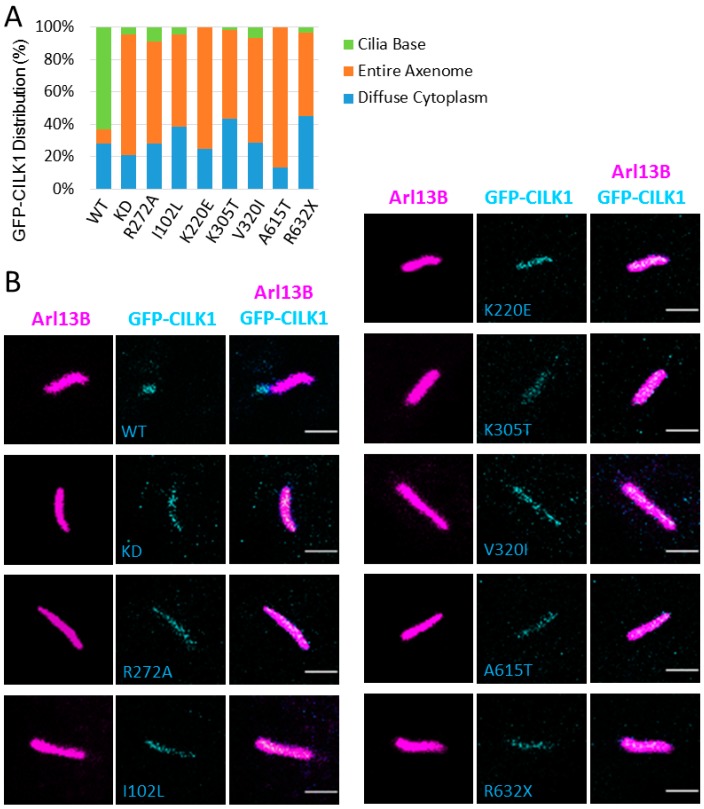
CILK1 variants redistribute along the entire axoneme of the primary cilium. NIH-3T3 cells were transiently transfected with GFP-CILK1 (wild type (WT); kinase-dead (KD); disease variants) and immunostained for the cilium with anti-Arl13B. Shown here are (**A**) the subcellular distribution patterns of GFP-CILK1 WT and disease variants; (**B**) confocal microscopy images of GFP-CILK1 (cyan), Arl13B (magenta), and their overlay. Pseudocolors were used here to facilitate visualization. Scale bar, 2 µm.

We postulated that disease-associated variants of CILK1 may suffer from mis-localization of CILK1. To test our hypothesis, we analysed intracellular localization by expressing GFP-CILK1 WT and CILK1 variants in NIH-3T3 cells. In the majority (>60%) of cells expressing WT GFP-CILK1, GFP signals are mostly restricted to the basal body, with minor amount localized in the cilium and cytoplasm (Figure 5). In contrast, all the GFP-CILK1 variants exhibited predominant distribution along the axoneme, shown by the image overlay between GFP-CILK1 and Arl13B (Figure 5). There was a dramatic reduction in the fraction of GFP-CILK1 variants at the basal body. Overall, CILK1 variants show a significant change in localization relative to WT, from the cilium base to the cilium axoneme.

## 4. Discussion

The primary cilium is conserved across species as a cellular sensory antenna and its genesis and size is controlled by RCK protein kinases. In humans, the RCK family kinase CILK1 plays a pivotal role in restricting ciliogenesis and cilia length. This study explored how mutations found in JME affect the functions of CILK1. Recently, we showed that both kinase activity and ciliary targeting involving the non-catalytic C terminal domain are essential for CILK1 to suppress ciliogenesis [12]. In the current study, we found that some CILK1 mutations in the non-catalytic CTD do not compromise catalytic activity but eliminate inhibition of ciliogenesis. These separation of function mutations show that CILK1 kinase activity alone is not sufficient for ciliary functions. A common phenotype exhibited by all CILK1 JME variants is their mislocalization from the basal body into the primary cilium. Given that the CTD is critical for ciliary targeting and substrate recognition, we speculate that the pathogenic JME variants in the CTD are mislocalized in the primary cilium and, therefore, fail to compartmentalize with substrates at the cilium base. We cannot exclude the possibility that mislocalization of CILK1 variants outside the primary cilium, such as in the cytosol, also contributes to their phenotype in the primary cilium.

Previously, we reported that the unstructured CTD is required for CILK1 targeting to the cilium base [12]. Here, our data indicate that JME variants in the kinase domain that inactivate CILK1 mislocalize from the cilium base to the axoneme. This result suggests that the catalytic activity of CILK1 is also necessary for its localization to the cilium base, which is consistent with prior observations that inactivating mutations in the kinase domain of CILK1 found in ciliopathies cause CILK1 mislocalization in the primary cilium [13,22,23,25]. It remains to be determined how the CTD and kinase activity both contribute to CILK1 targeting and localization in the basal body. One possible explanation is that CILK1 binding to, and phosphorylation of, a substrate at the cilium base is required for its anchoring to the basal body; therefore, CILK1 localization requires both the CTD which is critical for substrate binding and its kinase activity. It is worth pointing out that a previous report showed staining for endogenous CILK1 only at the tip of the cilium in mouse embryonic fibroblast (MEF) cells [13]. We note these cells were double stained for pericentrin, which may have obscured detection of CILK1 at the cilia base. Whether CILK1 variants exhibit cell type-specific patterns of mislocalization in the primary cilium is an interesting point for future investigation.

The mechanism underlying the effect of CILK1 variants on the length and the number of cilia is still unclear. It may be an effect mediated via changes in ciliary microtubule assembly or IFT. In Figures S4 and S5, we provided some preliminary data exploring the effect of GFP-CILK1 variants A615T and K220E on ciliary localization of intraflagellar transport components IFT88 (intraflagellar protein 88) and KIF3A, respectively. In cells expressing GFP-CILK1 WT, IFT88 shows two major ciliary distribution patterns: base enrichment and base/tip enrichment. Neither of these two GFP-CILK1 variants causes any significant changes in the pattern of IFT88 ciliary localization (Figure S4). In GFP-CILK1 WT cells, KIF3A is distributed either along the entire axoneme or enriched at the base. While GFP-CILK1 K220E variant appears to exert little effect on KIF3A ciliary localization, GFP-CILK1 A615T variant causes KIF3A enrichment at both the tip and the base of cilia in a fraction of GFP-positive cells (Figure S5). These preliminary results suggest potential variant-specific changes in certain IFT components. Given these are qualitative data, quantitative measurement of IFT in CRISPR/Cas9-engineered CILK1 variants knock-in cells are necessary for further evaluation of the impact of CILK1 variants on ciliary transport. We also want to point out that potential changes in the cell state, including proliferation and differentiation, are alternative mechanisms by which CILK1 variants can affect cilia length and number.

Previous studies have shown CILK1 phosphorylation of KIF3A at Thr-672 in vitro and in vivo and suggested that KIF3A may be the CILK1 substrate that mediates its downstream effect on anterograde IFT, which is critical for cilia formation and growth [12,13]. An intriguing observation we made in this study is that CILK1 variants in the CTD that retain the ability to phosphorylate KIF3A-Thr672 produce defects in both cilia formation and cilia length. This clearly indicates that KIF3A is not the sole CILK1 substrate responsible for the effects on ciliary phenotype. A significant challenge for future study is to identify other CILK1 substrates at the cilium base that are related to its restrictive control of ciliogenesis and cilia length.

## 5. Conclusions

In this study, we investigated the impact of JME-related pathogenic CILK1 variants on the primary cilium. We concluded that these variants lose the ability to suppress cilia length but gain function in promoting ciliogenesis. Our data suggest that CILK1 mislocalization affects the primary cilium, but not CILK1 phosphorylation of KIF3A. Our findings implicate that loss of restriction on ciliogenesis and cilia length may contribute to the cellular mechanism underlying the pathogenesis of JME.

## Supplementary Materials

The following are available online at https://www.mdpi.com/2073-4409/9/3/694/s1, Figure S1: Expression of GFP-CILK1 variants in NIH-3T3 cells; Figure S2: Quantification of green fluorescence intensity; Figure S3: Effects of CILK1 variants on cilia length; Figure S4: Ciliary localization of IFT88 in GFP-CILK1-positive NIH-3T3 cells; Figure S5: Ciliary localization of KIF3A in GFP-CILK1-positive NIH-3T3 cells.
