# Supplementary material for: Functional Alterations in Ciliogenesis-Associated Kinase 1 (CILK1) that Result from Mutations Linked to Juvenile Myoclonic Epilepsy
Source: Cells. 2020 Mar 12;9(3):694. doi: 10.3390/cells9030694 (PMC7140639; doi:10.3390/cells9030694)
Supplement: Supplementary file 1 [file cells-09-00694-s001.pdf]

Figure S1

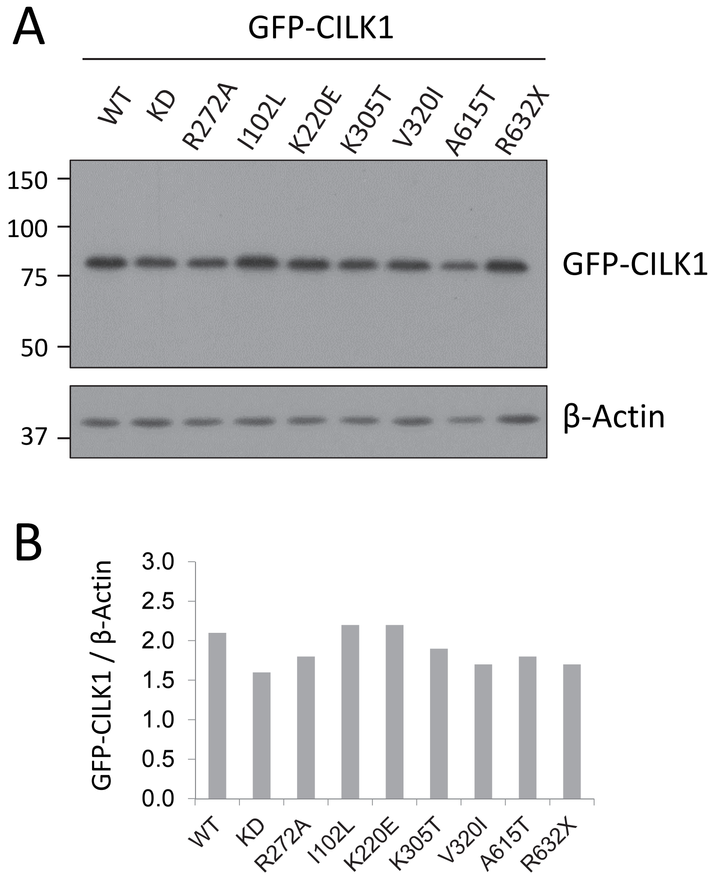

Figure S1. Expression of GFP-CILK1 variants in NIH-3T3 cells. (A) Equal amount of total proteins from whole cell extracts were Western blotted with GFP and β-Actin antibodies; (B) GFP-CILK1 signals were quantified using ImageJ and normalized against β-Actin signals on the Western blot.

Figure S2

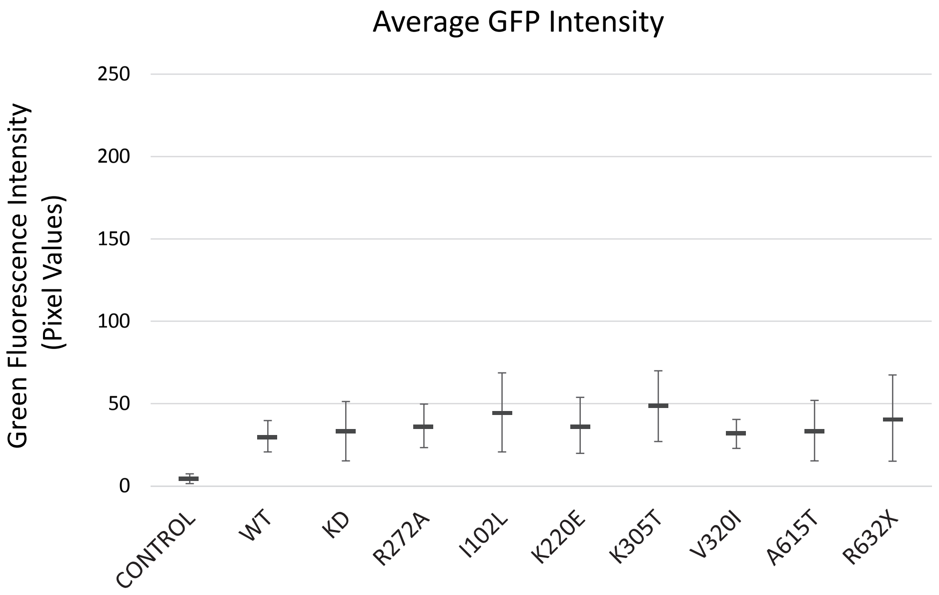

Figure S2. For GFP-positive cells, the average pixel intensity between 0 (no signal) to 255 (maximum signal) was measured of GFP channel to create an averaged GFP intensity score. Cells that highly overexpressed GFP at pixel values above 120 were excluded from analysis. Control cells expressed GFP at pixel values lower than 5. Cells expressing moderate levels of GFP at pixel values in the range from 20 to 70 were selected for analysis of primary cilia.

Figure S3

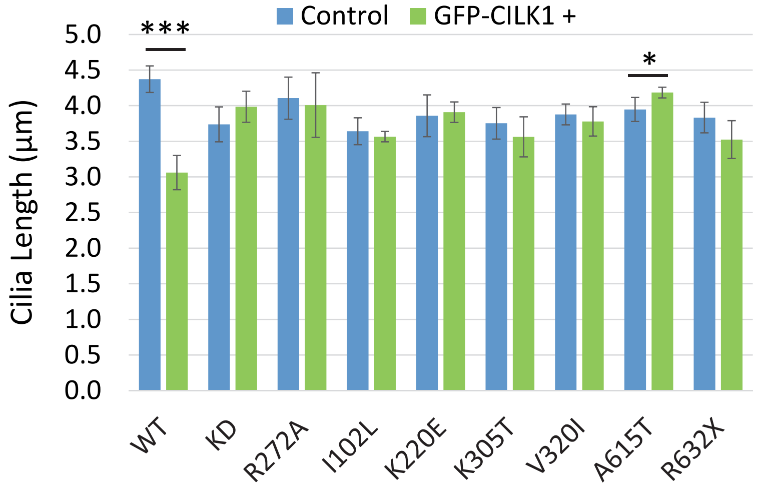

Figure S3. Effects of CILK1 variants on cilia length. GFP-CILK1 (wild type, WT; kinase-dead, KD; disease variants) were expressed in NIH-3T3 cells. The primary cilium was immunostained with rabbit anti-Arl13B followed by Alexa Fluor 594-conjugated goat anti-rabbit. Cilia length of GFP-CILK1 positive cells and control cells in the same culture plate was measured on confocal immunofluorescence images using ImageJ. Shown are mean ± SD, ***$P<0.001$, *$P<0.05$.

Figure S4

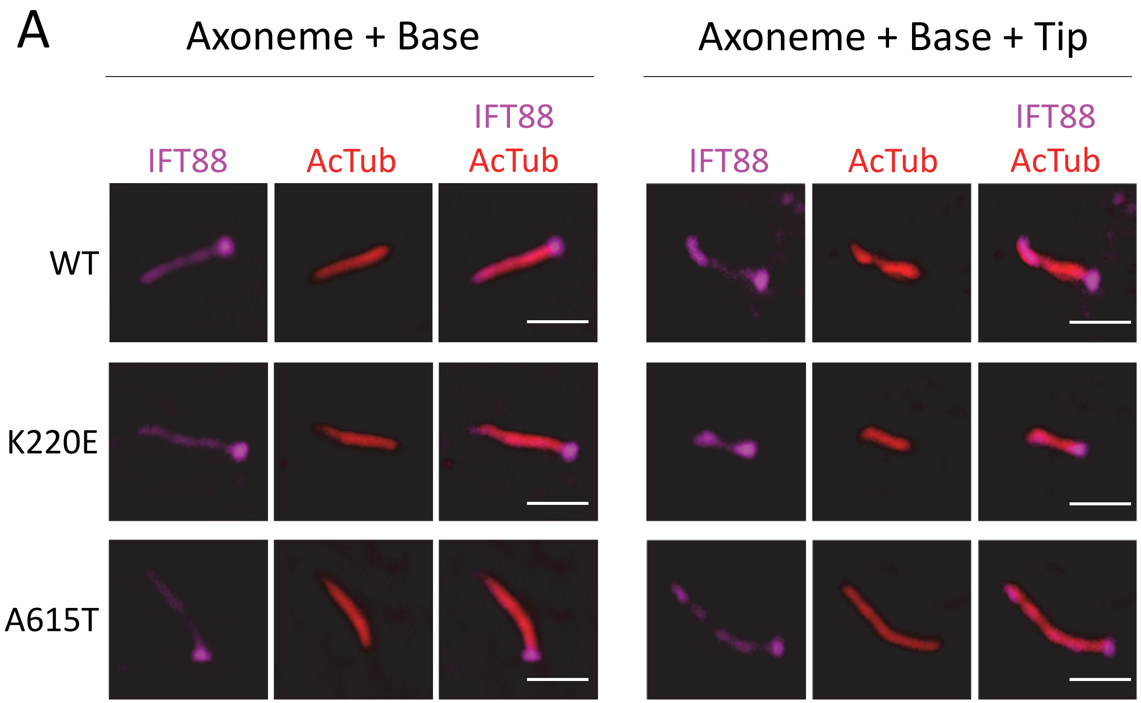

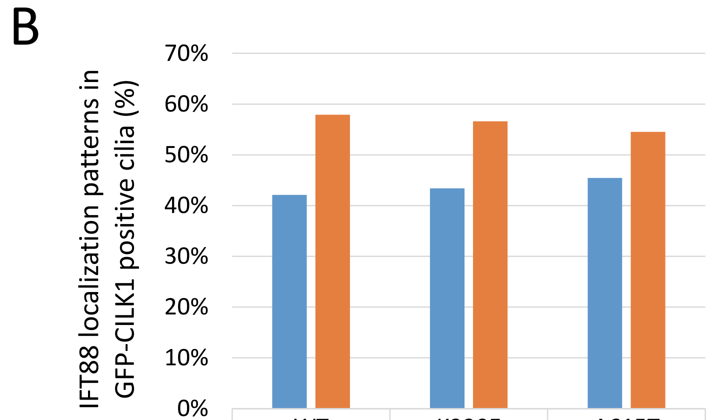

	WT	K220E	A615T
Axoneme + Base	42%	43%	45%
Axoneme + Base + Tip	58%	57%	55%

Figure S4. Ciliary localization of IFT88 in GFP-CILK1-positive NIH-3T3 cells. (A) NIH-3T3 cells were transfected with GFP-CILK1 WT, K220E, or A615 variant and co-immunostained with anti-IFT88 conjugated to Alexa Fluor 647 and anti-Acetylated Tubulin conjugated to Alexa Fluor 594. Shown are confocal immunofluorescence images of IFT88, Acetylated-Tubulin (AcTub) and their overlay in GFP-CILK1-positive cells. Scale bar, 2µm. (B) Summary of ciliary localization patterns of IFT88 in GFP-positive cells expressing WT, K220E, and A615T GFP-CILK1.

Figure S5

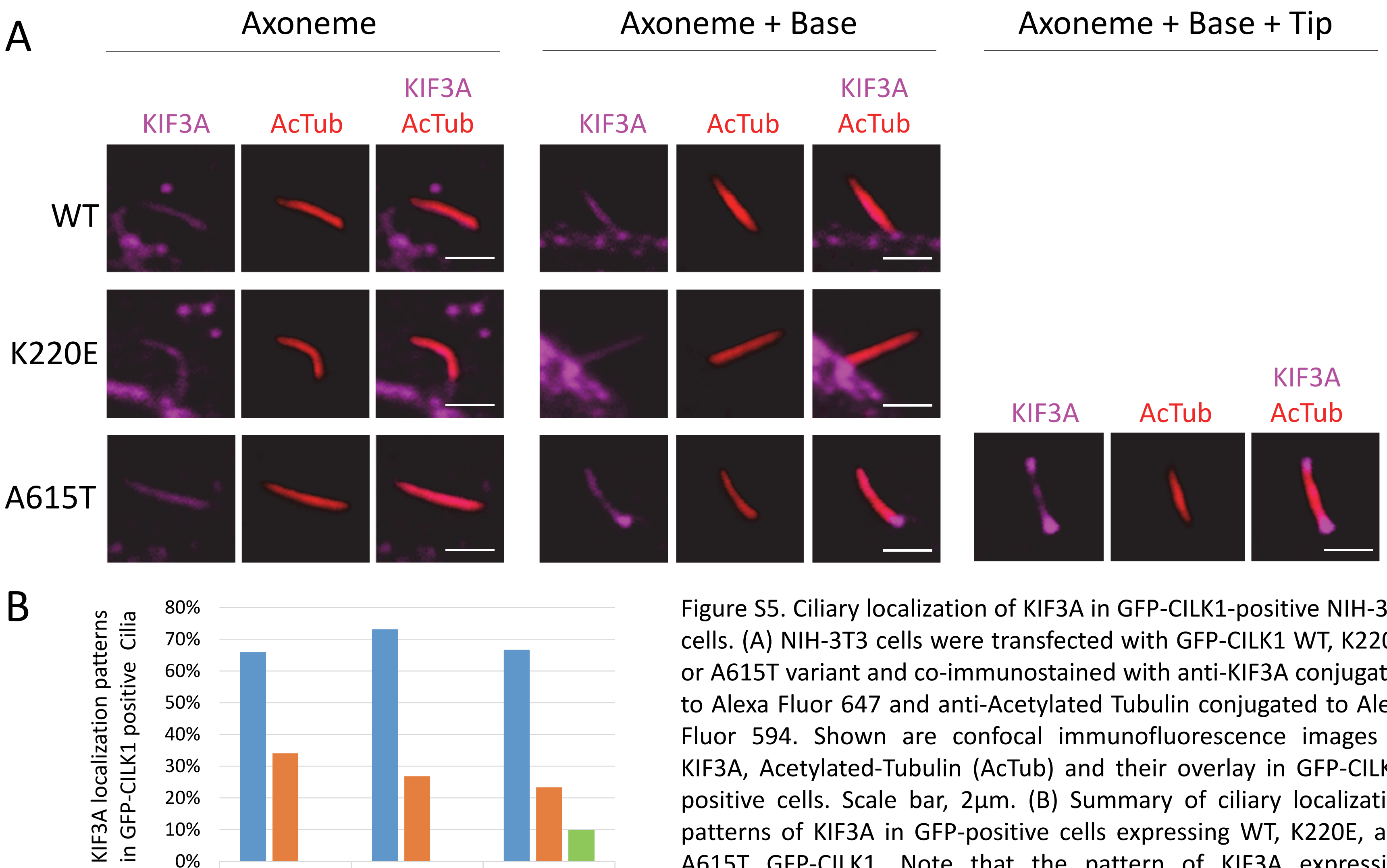

	WT	K220E	A615T
Axoneme	66%	73%	67%
Axoneme + Base	34%	27%	23%
Axoneme + Base + Tip	0%	0%	10%

Figure S5. Ciliary localization of KIF3A in GFP-CILK1-positive NIH-3T3 cells. (A) NIH-3T3 cells were transfected with GFP-CILK1 WT, K220E, or A615T variant and co-immunostained with anti-KIF3A conjugated to Alexa Fluor 647 and anti-Acetylated Tubulin conjugated to Alexa Fluor 594. Shown are confocal immunofluorescence images of KIF3A, Acetylated-Tubulin (AcTub) and their overlay in GFP-CILK1-positive cells. Scale bar, 2µm. (B) Summary of ciliary localization patterns of KIF3A in GFP-positive cells expressing WT, K220E, and A615T GFP-CILK1. Note that the pattern of KIF3A expression enriched at both the ciliary base and tip was only observed in a small fraction of GFP-CILK1-positive cells expressing the A615T variant as compared with either WT or the K220E variant.
